# Changes in miRNA Gene Expression during Wound Repair in Differentiated Normal Human Bronchial Epithelium

**DOI:** 10.1155/2018/9093785

**Published:** 2018-09-05

**Authors:** Beata Narożna, Wojciech Langwiński, Claire Jackson, Peter M. Lackie, John W. Holloway, Zuzanna Stachowiak, Monika Dmitrzak-Węglarz, Aleksandra Szczepankiewicz

**Affiliations:** ^1^Laboratory of Molecular and Cell Biology, Department of Pediatric Pulmonology, Allergy and Clinical Immunology, Poznan University of Medical Sciences, Poznan, Poland; ^2^Clinical and Experimental Sciences, Faculty of Medicine, University of Southampton, Southampton, UK; ^3^Human Development and Health, Faculty of Medicine, University of Southampton, Southampton, UK; ^4^Department of Psychiatric Genetics, Poznan University of Medical Sciences, Poznan, Poland

## Abstract

**Purpose:**

Airway epithelium acts as a protective barrier against the particles from the inhaled air. Damage to the epithelium may result in loss of the barrier function. Epithelial repair in response to injury requires complex mechanisms, such as microRNA, small noncoding molecules, to regulate the processes involved in wound repair. We aimed to establish if the microRNA gene expression profile is altered during the airway epithelial repair in differentiated cells.

**Methods:**

miRNA gene expression profile during the wound closure of differentiated normal human bronchial epithelium (NHBE) from one donor was analysed using quantitative real-time PCR. We have analysed the expression of 754 genes at five time points during a 48-hour period of epithelium repair using TaqMan Low Density Array.

**Results:**

We found out that 233 miRNA genes were expressed in normal human bronchial epithelium. Twenty miRNAs were differentially expressed during the wound repair process, but only one (miR-455-3p) showed significance after FDR adjustment (*p* = 0.02). Using STEM, we have identified two clusters of several miRNA genes with similar expression profile. Pathway enrichment analysis showed several significant signaling pathways altered during repair, mainly involved in cell cycle regulation, proliferation, migration, adhesion, and transcription regulation.

**Conclusions:**

miRNA expression profile is altered during airway epithelial repair of differentiated cells from one donor in response to mechanical injury *in vitro*, suggesting their potential role in wound repair.

## 1. Introduction

Upper respiratory airways are lined by pseudostratified columnar epithelium, composed of ciliated cells, goblet cells, and basal cells. The airway epithelium functions as a barrier that protects the lungs from inhaled pathogens and environmental particles [1]. The combined function of ciliated and secretory cells is responsible for maintaining efficient mucociliary clearance. Respiratory epithelium also plays a crucial role in the integration of innate and adaptive immune responses [2].

Damage to the airway epithelium might result in loss of barrier function and mucosal activation [3]. Physiological repair of the epithelium occurs in a series of overlapping processes. Studies in animals have shown that damage to the epithelium is rapidly restored by migration of remaining cells into the wound, followed by proliferation and differentiation [4, 5]. Epithelial injury and impaired repair underlie remodelling of the airways and underlie the development of respiratory diseases such as asthma [6]. Studies on cell cultures from asthmatic patients have shown that increased Th2 cell numbers and higher production of IL4 and IL14 cytokines result in decreased barrier integrity [7, 8].

MicroRNAs are small, noncoding RNA molecules that regulate gene expression either by degrading the target mRNA or by acting as translational enhancers or repressors. Several studies have shown their importance as regulators in development [9], proliferation [10], differentiation [11], apoptosis [12], immune response [13], and stem cell division [14]. However, the exact role of miRNA genes in the process of airway epithelial wound repair is still unclear. We hypothesized that miRNAs might coordinate the regulation of genes involved in the wound repair process. Our previous study has shown that microRNA profile was altered during bronchial epithelial repair in cells grown in monolayer (16HBE14o- cell line) [15]. The purpose of this study was to investigate whether changes in miRNA expression profile are also observed in the differentiated epithelium during repair. Since the epithelium injury and its abnormal repair are crucial in the pathogenesis of chronic airway diseases, such as asthma, the development of targeted therapy using inhibitors or synthetic miRNAs could be a breakthrough in the treatment of these diseases.

## 2. Materials and Methods

### 2.1. Cell Culture and Wounding Assays

NHBE cell line (Lonza) from one nonsmoking donor was cultured in a bronchial epithelium growth medium (BEBM medium with SingleQuot Kit Supplements and Growth Factors, Lonza) on 75 cm^2^ until 85% confluent. Subsequently, the cells were passaged (3 × 10^5^ cells on 1.2 cm^5^) onto collagen-coated transwell inserts in a 12-well culture plate (Costar, Corning) and were cultivated until confluent. Afterwards, the basolateral chamber medium was switched to the air-liquid interface (ALI) (1 : 1, BEBM : DMEM, 3.5 g/L D-glucose with SingleQuots), supplemented with 100 nM retinoic acid (Sigma-Aldrich). The apical surface of the cell culture was then exposed to air. The medium was replaced three times per week. Any mucus that has appeared on the top of cells was systematically removed. Cilia were observed 3–4 weeks posttransition to ALI. Differentiated cells were scratched by P200 Gilson pipette tip. Cell debris was removed, and a fresh medium was added to the basolateral chamber.

### 2.2. Time-Lapse Microscopy

Time-lapse images were collected for 48 hours until complete wound closure at 15-minute intervals on the Olympus IX81 microscope, using xCellence software. The chamber was maintained at 36 ± 1°C and 5% CO_2_ atmosphere. The wound area and the ongoing repair process were analysed using ImageJ software [16]. Wound recovery was calculated by tracing the new wound edge at each time interval and comparing the wound width to that of the original wound edge at the beginning of the experiment.

### 2.3. RNA Isolation

RNA was isolated from NHBE cells with the use of miRCURY RNA Isolation Kit—Cell and Plant (Exiqon), according to the manufacturer's instructions. Three biological replicates were collected at five consecutive time points: baseline (before wounding), 8, 16, 24, and 48 h after wounding. The amount of starting material was around 0.7 × 10^5^ cells per well. Samples were stored at −70°C until the microarray experiment could be performed. The total RNA concentration was measured using NanoDrop 2000 spectrophotometer.

### 2.4. MicroRNA Profiling

For profiling, we used the TaqMan Array Human MicroRNA Cards A and B that contain 754 human microRNAs. Reverse transcription was done using Megaplex Primer Pools (Human Pools A v.2.1 and B v.3.0, Thermo Fisher Scientific) and TaqMan® MicroRNA Reverse Transcription Kit (Thermo Fisher Scientific) according to the manufacturer's protocol. TaqMan Universal PCR Master Mix, No AmpErase UNG (Thermo Fisher Scientific) was combined with diluted cDNA and loaded into TaqMan Array Human MicroRNA Card A v.2.0 or Card B v.3.0 and centrifuged. Quantitative real-time PCR was conducted in the 7900HT Fast Real-Time PCR System (Applied Biosystems). The reaction was performed in triplicate for each sample. Raw expression data were acquired from SDS 2.4 software (Applied Biosystems) and further analysed with RQ Manager 1.2.1 (Applied Biosystems). The comparative analysis of obtained datasets between baseline and each time point was accomplished in DataAssist v.3.01 software (Applied Biosystems). Undetermined values were considered as equal to the maximum allowable Ct value (37). In order to reduce background noise, we have excluded miRNAs that were not expressed in 90% of the samples. Outliers were removed from the analysis after applying a refined Grubbs' outlier test. Each Ct value of target miRNA was normalized against the mean of the selected endogenous control, U6 snRNA-001973. Normalized miRNA expression was assessed against the baseline using the 2^−ΔCt^ method. All up- or downregulated miRNAs with a fold expression ≥2 and *p* value < 0.05 were considered to be differentially expressed. The *p* values were adjusted for multiple tests using the Benjamini-Hochberg false discovery rate (FDR).

### 2.5. Cluster Analysis

The clusters of miRNAs with similar expression profile over time were identified by cluster analysis in STEM (Short Time series Expression Miner) software available at http://www.cs.cmu.edu/~jernst/stem/ [17].

### 2.6. Target Genes and Pathways Prediction

We have performed pathway enrichment analysis to identify common biological pathways for miRNAs with similar expression profile. For each miRNA, we have identified the best predicted mRNA target genes using miRNA BodyMap tool (http://www.mirnabodymap.org). All available prediction algorithms were used: DIANA, PITA, TargetScan, RNA22 (3'UTR), RNA22 (5'UTR), TargetScan_cons, MicroCosm, miRDB, TarBase, and miRecords. However, to minimise the target prediction noise, we included the target genes predicted by at least four of these algorithms.

The list containing the best predictions was then analysed with the use of the Database for Annotation, Visualization and Integrated Discovery (DAVID) v.6.7 [18, 19], which allowed identifying BioCarta and KEGG pathways [20], enriched functional-related gene groups, and biological themes.

### 2.7. Mir-455-3p Targets

Based on available literature, target prediction results, and gene function, we have chosen the genes encoding isoforms of transforming growth factor *β* (*TGFβ1*, *TGFβ2*, and *TGFβ3*) and their receptors (*TGFβR1*, *TGFβR2*, and *TGFβR3*) for the gene expression analysis. Reverse transcription was done with the use of GoScript™ Reverse Transcription System (Promega), and resulting cDNA was analysed in quantitative real-time PCR using dye-based qPCR Master Mix (Promega) and set of specific primers spanning exon-exon junction (sequences are presented in Table 1). Each Ct value of target mRNA was normalized against an endogenous control, *PPIA*, which had shown to have the most stable expression in all samples tested (out of 10 potential endogenous controls).

## 3. Results

### 3.1. Epithelial Wound Repair Model

**Figure 1:**
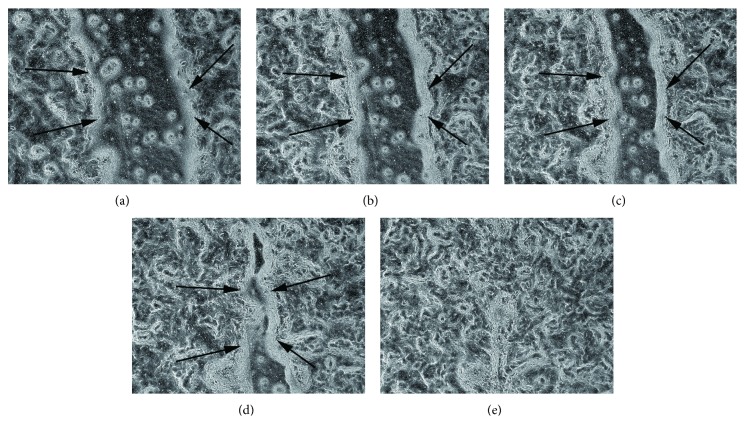
Representative images of wound repair at different time points: (a) 0 hrs, (b) 8 hrs, (c) 16 hrs, (d) 24 hrs, and (e) 48 hrs postwounding. *n* = 3 wells for each time point.

Based on the representative images from time-lapse microscopy, we have selected the following time points for miRNA profile analysis: the baseline immediately before injury (Figure 1(a)); 8 hours after wounding: the cells adjacent to the wound have initiated a response and have started to migrate, covering around 15% of the original wound area (SD 4.50, SEM 2.0) (Figure 1(b)); 16 hours after wounding: 35% of the wounded area has been covered by the cells (SD 4.24, SEM 1.89) (Figure 1(c)); 24 hours after wounding: 50% of the wounded area covered by cells (SD 3.43, SEM 1.54) (Figure 1(d)); the wounded area entirely covered by cells (Figure 1(e)). Cell death was not observed in the repairing areas, with the exception of the cells damaged by the mechanical scratch.

### 3.2. Altered miRNA Expression Profile during Epithelial Wound Repair

**Figure 2:**
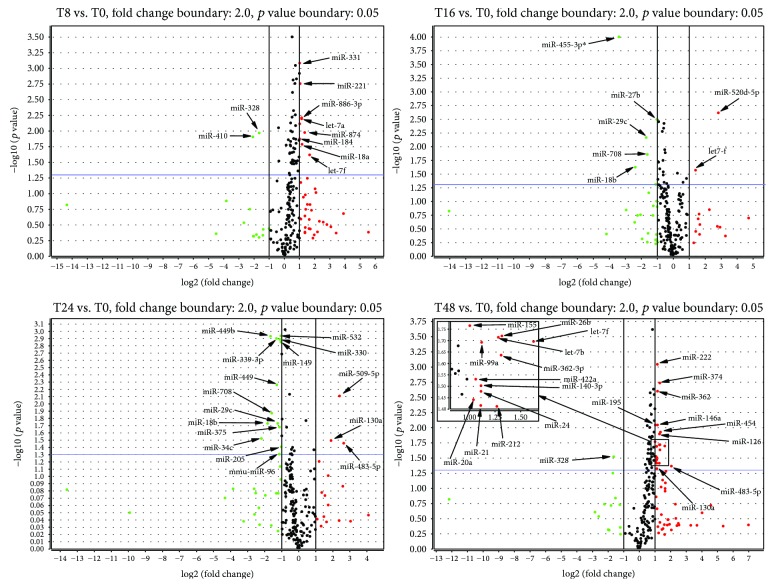
Volcano plots for different time points. T8: 8 h after wounding; T16: 16 h after wounding; T24: 24 h after wounding; T48: 48 h after wounding (reference: baseline; fold-change boundary: 2.0; *p* value boundary: 0.05; arrows are pointing out statistically significant results; ^∗^results remaining significant after FDR correction).

After normalization, we found out that 230 miRNAs from Card A and 3 miRNAs from Card B were expressed in normal bronchial epithelium. Analysis of miRNA expression has revealed a large number of genes with significantly increased or decreased expression at different time points (fold change above 2.0, *p* < 0.05).  Figure 2 shows volcano plots for each time point of wound repair (8, 16, 24, and 48 hours postwounding) compared to the baseline. After multiple testing correction (false discovery rate *p* value less than 0.05), we found significant changes in the expression for miR-455-3p (*p* = 0.02) 16 hours postwounding as compared to the baseline.

### 3.3. Cluster Analysis

**Figure 3:**
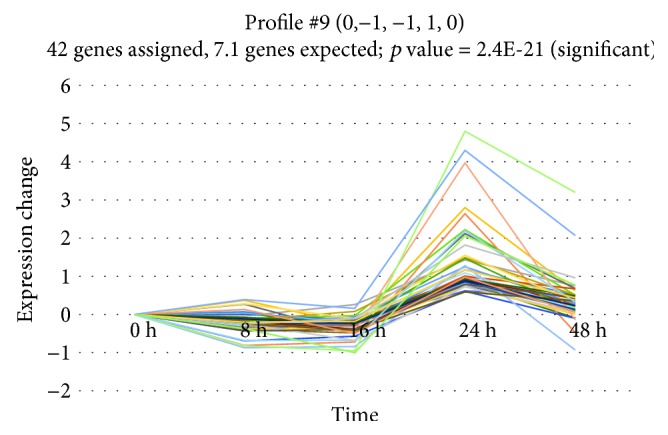
Profile 9 of differentially expressed miRNAs with similar expression pattern during wound repair.

**Figure 4:**
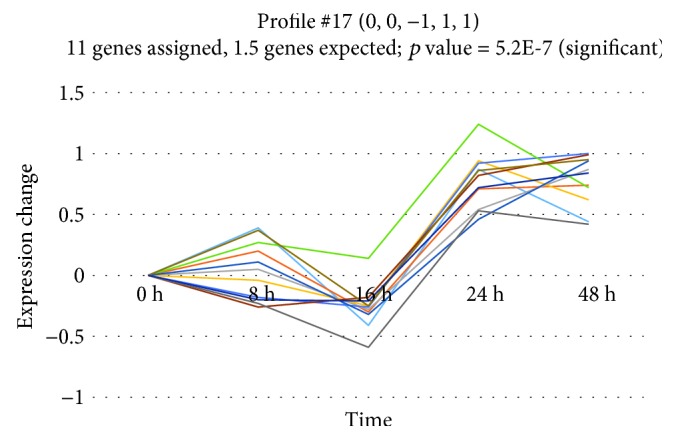
Profile 17 of differentially expressed miRNAs with similar expression pattern during wound repair.

To investigate if miRNA genes share a common expression profile during epithelial repair, we performed cluster analysis using the STEM algorithm. This calculation revealed that, out of 40 model profiles, two profiles (profiles 9 and 17) showed significant enrichment during repair (*p* = 2.4*E* − 21 and *p* = 5.2*E* − 7, respectively) (Figures 3 and 4). The list of miRNA genes for each profile can be found in Supplementary Materials (available here). Profile 9 groups 42 miRNA genes (12 of them differed significantly before FDR correction) and is characterised by a gradual increase in miRNA expression until 8 hours after wounding and decrease until 16 hours postwounding, followed by a significant increase at 24 hours of wound repair and decrease at 48 hours approximately to baseline. Profile 17, consisting of 11 genes (3 of them differed significantly before FDR correction), has a similar pattern until 24 hours, after which the decrease in expression is much smaller.

### 3.4. Pathway Enrichment Analysis

Our next step was to investigate whether miRNAs with the same expression patterns during airway epithelial repair may regulate target genes from the same biological pathways. Firstly, we have created a list of the best target mRNAs for each miRNA gene (8292 genes in total for profile 9 and 173939 genes for profile 17) and analysed these genes with DAVID online database. We have found several significantly enriched pathways for each profile that remained significant after correction for multiple testing (Tables 2 and 3), including the MAPK signaling pathway (regulation of transcription and translation, inflammation, cell stress response, differentiation, division, proliferation, metabolism, motility, and apoptosis), PI3K-Akt signaling pathway (metabolism, growth, proliferation, survival, transcription, and protein synthesis), focal adhesion (cell motility, survival, proliferation, differentiation, and regulation of gene expression), regulation of actin cytoskeleton (cell motility and shape regulation, proliferation, secretion, phagocytosis, and cell communication), ErbB signaling pathway (cell proliferation, differentiation, motility, and survival), and neurotrophin signaling pathway.

### 3.5. Mir-455-3p Targets

**Figure 5:**
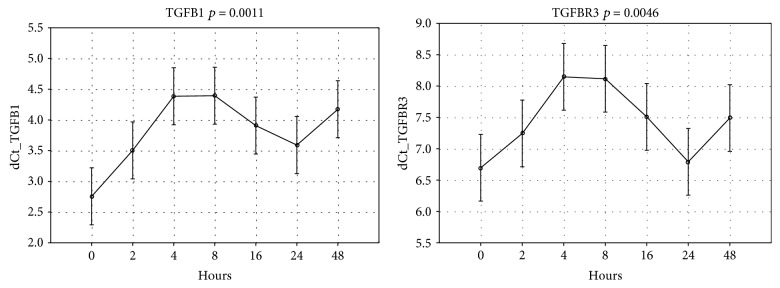
ANOVA analysis of TGF-*β*1, TGF-*β*3, TGF-*β*R1, TGF-*β*R2, and TGF-*β*R3 changes in mRNA expression.

Analysis of six targets from TGF*β* family showed that the mRNA expression of two of them, *TGF-β1* and *TGF-βR3,* showed significantly altered expression upon repair (Figure 5). Their expression profiles during wound repair suggest negative correlation with the expression profile of miR-455-3p: the expression of these two genes significantly increased 8 hours postwounding, while the miRNA expression decreased, whereas between 8 and 24 hours of repair, the expression of *TGF-β1* and *TGF-βR3* decreased and miR-455-3p expression increased. The expression of the other four genes (*TGFβ2*, *TGF-β3*, *TGF-βR1*, and *TGF-βR2)* was not significantly changed during repair (*p* > 0.05).

## 4. Discussion

The most important finding of our study is the involvement of miRNA genes in the *in vitro* wound repair of the differentiated airway epithelium from one donor. That corresponds to the results of our previous research [15], reporting changes in miRNA expression in undifferentiated cell line (16HBE14o-). Our recent findings [21] have also confirmed the importance of miRNA during this process: silencing of *DICER* and *DROSHA*, the enzymes crucial for RNA interference, resulted in significantly delayed wound repair. Profiling analysis of both cell types (undifferentiated and pseudostratified epithelium) showed some similarities between the expressions of several miRNA genes, suggesting their importance in the fundamental repair processes.

Epithelial wound repair in the airways *in vivo* consists of cell spreading and migration within 12–24 hours postwounding. The proliferation process begins by 15–24 hours and can continue for days to weeks. While *in vitro* model mimics *in vivo* situation, it happens in a much shorter time frame. What is interesting, the NHBE cells require more time than undifferentiated 16HBE14o- until complete wound closure. Since most of the cells are ciliated, they need to dedifferentiate first. Therefore, the migration and proliferation observed in the first hours of the repair are most likely caused by the involvement of basal cells, suggesting that the ALI model may be more appropriate to study the wound repair.

The cluster analysis of time series miRNA expression data from one donor revealed distinct expression patterns of miRNA gene clusters during wound repair. The relationship between these miRNAs, their putative targets, and changes in individual protein levels during airway epithelial wound repair needs to be validated in future studies. However, the observed changes in the expression of the whole cluster of miRNA genes seem to be a consequence of the injury. For both profiles, we identified several signaling pathways responsible for the regulation of wound repair, some of which were also significant during the repair in undifferentiated 16HBE14o- cell line: the ErbB signaling pathway, MAPK signaling pathway, pathways in cancer, and neurotrophin signaling pathway [15].

Only the expression of one miRNA gene, miR-455-3p, was found to be significantly altered during the wound repair. MiR-455-3p was previously described as a tumour suppressor in several human cancers [22–24]. Gao et al. [25] found downregulated expression of this gene in non-small-cell lung cancer (NSCLC) tissues that correlated with poor prognosis of NSCLC patients. Furthermore, they revealed that miR-455-3p inhibited cell proliferation and migration *in vitro* via direct targeting *HOXB5*, a member of the HOX gene family. Decreased expression of miR-455-3p in our study complies with the previous findings and possibly enables to switch on genes involved in cell proliferation and migration required during wound repair.

Martinez-Anton et al. [11] have studied the differences in miRNA expression changes during differentiation of normal human bronchial cells. MiR-455-3p expression was significantly downregulated during differentiation; we have also observed its decreased expression during the epithelial wound repair. Their confirmatory experiments also showed that MUC1 mRNA is a target gene of miR-455-3p. Since MUC1 encodes mucin responsible for protecting the cells from pathogens, decreased expression of miR-455 in our study suggests that the injury results in the loss of barrier function.

TGF-*β* is a multifunctional cytokine, involved in cell growth, inflammation, and repair [26]. Ong et al. have discovered that miR-455-3p was induced by TGF-*β* in lung fibroblast, and immunoprecipitation of Ago2 revealed enrichment of miR-455-3p targets related to TGF-*β* and/or the Wnt pathway [27]. Our results showed that the expression profiles of TGF-*β*1 and TGF-*β*R3 correspond to the changes in the expression of miR-455-3p, suggesting this miRNA is possibly involved in posttranscriptional regulation of these two TGF genes.

The main limitation of our study was the use of biological replicates from only one donor. While changes in miRNA expression during wound repair were observed in cells from one donor and in other airway epithelium cell line, we cannot state for certain that this will be the case for other donors, due to donor-to-donor variability. Additional experiments including more donors, as well as the confirmation of the miR-455-3p involvement in *TGF-β1* and *TGF-βR3* expression regulation, are planned in future research.

In conclusion, we showed that the expression of multiple miRNAs is altered during airway epithelial repair in differentiated cells from one donor, suggesting their importance in the regulation of this process. We also observed two common expression profiles for several miRNA genes, and in silico analysis of predicted mRNA target genes has shown that they coordinate signaling pathways involved in the wound repair. We also found out that the *TGF* gene family is a possible target of miRNA genes altered upon epithelial repair, in particular miR-455-3p. However, further experiments on cells from more donors are required to investigate the exact role of identified miRNAs and their targets in epithelial repair.

## Figures and Tables

**Table 1:** Sequences of the primers used for target gene expression analysis.

Gene	Direction	Sequence
TGFB1	F	TTCAACACATCAGAGCTCC
	R	GCTGTATTTCTGGTACAGCT
TGFB3	F	CAAATTCAAAGGCGTGGAC
	R	ATTAGATGAGGGTTGTGGTG
TGFBR1	F	GAATCCTTCAAACGTGCTG
	R	TCATGAATTCCACCAATGGA
TGFBR2	F	GCTGTATGGAGAAAGAATGAC
	R	CAGAATAAAGTCATGGTAGGG
TGFBR3	F	TGATAATGGATTTCCGGGAG
	R	CTGCAATTAAACACCACGA
PPIA	F	AGACAAGGTCCCAAAGAC
	R	ACCACCCTGACACATAAA

**Table 2:** The results of pathway analysis of predicted target genes for profile 9 in DAVID database.

Category	Pathway	Enrichment score	No. of genes^∗^	*p*	*p* (FDR corrected)
KEGG	Pathways in cancer	1.4	259	5.70*E* − 17	1.40*E* − 13
KEGG	PI3K-Akt signaling pathway	1.4	221	1.90*E* − 12	2.60*E* − 09
KEGG	MAPK signaling pathway	1.5	170	7.80*E* − 12	1.00*E* − 08
KEGG	Focal adhesion	1.5	141	3.40*E* − 11	4.50*E* − 08
KEGG	Axon guidance	1.6	92	1.50*E* − 09	2.00*E* − 06
KEGG	Ras signaling pathway	1.4	146	7.30*E* − 09	9.70*E* − 06
KEGG	cGMP-PKG signaling pathway	1.5	111	3.90*E* − 08	5.20*E* − 05
KEGG	Rap1 signaling pathway	1.4	135	4.50*E* − 08	6.00*E* − 05
KEGG	Proteoglycans in cancer	1.4	129	7.00*E* − 08	9.40*E* − 05
KEGG	FoxO signaling pathway	1.5	92	1.00*E* − 07	1.40*E* − 04
KEGG	Regulation of actin cytoskeleton	1.4	133	3.10*E* − 07	4.10*E* − 04
KEGG	Signaling pathways regulating pluripotency of stem cells	1.5	94	3.70*E* − 07	4.90*E* − 04
KEGG	T cell receptor signaling pathway	1.6	73	3.90*E* − 07	5.30*E* − 04
KEGG	Adrenergic signaling in cardiomyocytes	1.5	97	5.00*E* − 07	6.60*E* − 04
KEGG	Melanoma	1.7	54	5.00*E* − 07	6.70*E* − 04
KEGG	cAMP signaling pathway	1.4	125	6.50*E* − 07	8.70*E* − 04
KEGG	Glutamatergic synapse	1.5	78	1.40*E* − 06	1.90*E* − 03
KEGG	Wnt signaling pathway	1.4	91	1.90*E* − 06	2.60*E* − 03
KEGG	HTLV-I infection	1.3	154	2.10*E* − 06	2.80*E* − 03
KEGG	Dopaminergic synapse	1.5	85	2.90*E* − 06	3.90*E* − 03
KEGG	Renal cell carcinoma	1.7	49	3.00*E* − 06	3.90*E* − 03
KEGG	Neurotrophin signaling pathway	1.5	80	4.70*E* − 06	6.30*E* − 03
KEGG	TNF signaling pathway	1.5	72	5.70*E* − 06	7.60*E* − 03
KEGG	ErbB signaling pathway	1.5	61	7.30*E* − 06	9.80*E* − 03
KEGG	Sphingolipid signaling pathway	1.4	79	1.10*E* − 05	1.50*E* − 02
KEGG	Prostate cancer	1.5	61	1.30*E* − 05	1.70*E* − 02
KEGG	Insulin signaling pathway	1.4	88	2.10*E* − 05	2.80*E* − 02
KEGG	Inflammatory mediator regulation of TRP channels	1.5	66	2.30*E* − 05	3.00*E* − 02
KEGG	Dorsoventral axis formation	2	24	2.30*E* − 05	3.10*E* − 02
KEGG	Pancreatic cancer	1.6	47	3.00*E* − 05	4.10*E* − 02
KEGG	Glioma	1.6	47	3.00*E* − 05	4.10*E* − 02
KEGG	Oxytocin signaling pathway	1.4	98	3.50*E* − 05	4.70*E* − 02
KEGG	Platelet activation	1.4	83	3.50*E* − 05	4.70*E* − 02
KEGG	Insulin resistance	1.4	71	3.60*E* − 05	4.80*E* − 02

^∗^Number of predicted target genes that are involved in the particular pathway.

**Table 3:** The results of pathway analysis of predicted target genes for profile 17 in DAVID database.

Category	Pathway	Enrichment score	No. of genes^∗^	*p*	*p* (FDR corrected)
KEGG	Pathways in cancer	1.5	128	4.40*E* − 07	5.90*E* − 04
KEGG	Proteoglycans in cancer	1.7	74	1.10*E* − 06	1.40*E* − 03
KEGG	MAPK signaling pathway	1.6	88	2.80*E* − 06	3.70*E* − 03
KEGG	Inflammatory mediator regulation of TRP channels	2	42	5.80*E* − 06	7.80*E* − 03
KEGG	PI3K-Akt signaling pathway	1.5	110	9.60*E* − 06	1.30*E* − 02
KEGG	GnRH signaling pathway	2	39	1.30*E* − 05	1.80*E* − 02
KEGG	Rap1 signaling pathway	1.6	72	3.20*E* − 05	4.30*E* − 02

^∗^Number of predicted target genes that are involved in the particular pathway.

## Data Availability

The access to the data can be made after contacting the Laboratory of Molecular and Cell Biology, Beata Narożna, e-mail: b.narozna@gmail.com.

## Supplementary Materials

Supplementary Materials MiRNA genes assigned to each expression profile during wound repair (values given for each time point represent expression change after normalization in STEM software).
