# Supplementary material for: Changes in miRNA Gene Expression during Wound Repair in Differentiated Normal Human Bronchial Epithelium
Source: Int J Genomics. 2018 Sep 5;2018:9093785. doi: 10.1155/2018/9093785 (PMC6145058; doi:10.1155/2018/9093785)
Supplement: Supplementary Materials — MiRNA genes assigned to each expression profile during wound repair (values given for each time point represent expression change after normalization in STEM software). [file 9093785.f1.docx]

**Additional file 1.** MiRNA genes assigned to each expression profile during wound repair (values given for each time point represent expression change after normalization in STEM software)

| **Gene** | **0 h** | **8 h** | **16 h** | **24 h** | **48 h** |
| --- | --- | --- | --- | --- | --- |
| **Profile 9 (P=2.4E-21)** | | | | | |
| hsa-let-7G-002282 | 0.00 | -0.21 | -0.25 | 0.98 | 0.33 |
| hsa-miR-125A-5P-002198 | 0.00 | -0.20 | -0.19 | 0.93 | 0.30 |
| hsa-miR-126-002228 | 0.00 | -0.04 | 0.26 | 1.43 | 0.75 |
| hsa-miR-130B-000456 | 0.00 | 0.37 | -0.27 | 2.80 | 0.63 |
| hsa-miR-135A-000460 | 0.00 | -0.43 | -0.17 | 0.62 | 0.47 |
| hsa-miR-138-002284 | 0.00 | -0.10 | -0.03 | 2.22 | 0.51 |
| hsa-miR-139-5P-002289 | 0.00 | 0.01 | -0.15 | 0.95 | 0.28 |
| hsa-miR-141-000463 | 0.00 | -0.12 | -0.16 | 0.97 | 0.29 |
| hsa-miR-150-000473 | 0.00 | 0.27 | -0.50 | 2.12 | 0.68 |
| hsa-miR-15B-000390 | 0.00 | -0.40 | -0.48 | 0.93 | 0.25 |
| hsa-miR-17-002308 | 0.00 | -0.34 | -0.16 | 0.73 | 0.21 |
| hsa-miR-181C-000482 | 0.00 | 0.06 | -0.42 | 0.84 | -0.09 |
| hsa-miR-186-002285 | 0.00 | 0.04 | -0.21 | 0.82 | 0.19 |
| hsa-miR-18B-002217 | 0.00 | -0.81 | -0.72 | 2.64 | -0.47 |
| hsa-miR-195-000494 | 0.00 | -0.27 | -0.31 | 1.02 | 0.08 |
| hsa-miR-197-000497 | 0.00 | -0.37 | -0.38 | 0.73 | 0.01 |
| hsa-miR-222-002276 | 0.00 | -0.24 | -0.06 | 1.25 | 0.28 |
| hsa-miR-223-002295 | 0.00 | -0.30 | -0.22 | 0.88 | 0.20 |
| hsa-miR-24-000402 | 0.00 | 0.07 | -0.29 | 1.17 | 0.51 |
| hsa-miR-25-000403 | 0.00 | -0.27 | -0.29 | 1.00 | 0.67 |
| hsa-miR-26A-000405 | 0.00 | -0.34 | -0.26 | 0.79 | 0.13 |
| hsa-miR-26B-000407 | 0.00 | -0.15 | 0.08 | 1.49 | 0.51 |
| hsa-miR-29C-000587 | 0.00 | -0.69 | -0.57 | 0.61 | -0.10 |
| hsa-miR-374-000563 | 0.00 | -0.17 | -0.20 | 1.47 | 0.10 |
| hsa-miR-422A-002297 | 0.00 | -0.29 | -0.11 | 1.09 | 0.15 |
| hsa-miR-423-5P-002340 | 0.00 | -0.22 | -0.44 | 3.97 | -0.14 |
| hsa-miR-449B-001608 | 0.00 | -0.25 | -0.68 | 0.73 | 0.06 |
| hsa-miR-454-002323 | 0.00 | 0.28 | -0.11 | 1.55 | 0.33 |
| hsa-miR-486-001278 | 0.00 | -0.87 | -0.84 | 1.28 | -0.93 |
| hsa-miR-505-002089 | 0.00 | -0.82 | -0.95 | 4.80 | 3.20 |
| hsa-miR-590-5P-001984 | 0.00 | -0.24 | -0.24 | 0.86 | 0.13 |
| hsa-miR-598-001988 | 0.00 | -0.19 | -0.38 | 0.90 | 0.42 |
| hsa-miR-625-002431 | 0.00 | -0.43 | -0.39 | 0.79 | 0.46 |
| hsa-miR-652-002352 | 0.00 | -0.25 | -0.41 | 0.59 | 0.36 |
| hsa-miR-660-001515 | 0.00 | -0.12 | -0.23 | 0.89 | 0.23 |
| hsa-miR-671-3P-002322 | 0.00 | -0.08 | -0.21 | 0.93 | 0.49 |
| hsa-miR-708-002341 | 0.00 | -0.68 | -0.67 | 2.19 | 0.33 |
| hsa-miR-890-002209 | 0.00 | 0.13 | -0.50 | 0.98 | 0.06 |
| hsa-miR-891A-002191 | 0.00 | -0.25 | -0.10 | 1.82 | 0.96 |
| hsa-miR-99A-000435 | 0.00 | -0.20 | -0.18 | 1.17 | 0.44 |
| mmu-miR-379-001138 | 0.00 | 0.39 | 0.16 | 4.30 | 2.07 |
| mmu-miR-495-001663 | 0.00 | -0.37 | -1.00 | 2.06 | 0.77 |
| **Profile 17** **(P=5.2E-7)** | | | | | |
| hsa-miR-148b-000471 | 0.00 | 0.39 | -0.41 | 0.87 | 0.44 |
| hsa-miR-200c-002300 | 0.00 | 0.20 | -0.30 | 0.71 | 0.74 |
| hsa-miR-210-000512 | 0.00 | 0.05 | -0.28 | 0.54 | 0.87 |
| hsa-miR-31-002279 | 0.00 | -0.04 | -0.25 | 0.94 | 0.62 |
| hsa-miR-340-002258 | 0.00 | -0.18 | -0.26 | 0.92 | 1.00 |
| hsa-miR-362-001273 | 0.00 | 0.27 | 0.14 | 1.24 | 0.72 |
| hsa-miR-425-5p-001516 | 0.00 | 0.11 | -0.32 | 0.46 | 0.94 |
| hsa-miR-429-001024 | 0.00 | -0.26 | -0.18 | 0.82 | 0.99 |
| hsa-miR-449-001030 | 0.00 | -0.23 | -0.59 | 0.53 | 0.42 |
| hsa-miR-484-001821 | 0.00 | 0.37 | -0.25 | 0.86 | 0.95 |
| hsa-miR-744-002324 | 0.00 | -0.20 | -0.21 | 0.72 | 0.84 |
